# Dynamic shunt flow alterations through patent foramen ovale during off-pump coronary artery bypass grafting induced by airway pressure changes: a case report

**DOI:** 10.1186/s40981-024-00748-7

**Published:** 2024-10-15

**Authors:** Ayano Honda, Koichi Yoshinaga, Yuji Hirasaki, Yusuke Iizuka, Yuji Otsuka

**Affiliations:** 1 Department of Anesthesiology and Critical Care Medicine, Jichi Medical University Saitama Medical Center, 1-847 Amanuma-Cho, Omiya-Ku, Saitama-Shi, Saitama, 330-8503 Japan; 2 Department of Anesthesiology and Critical Care Medicine, Jichi Medical University, 3311-1 Yakushiji, Shimotsuke-Shi, Tochigi, 329-0498 Japan; 3 Department of Anesthesiology, Saiseikai Utsunomiya Hospital, 911-1 Takebayashi-Cho, Utsunomiya-Shi, Tochigi, 321-0974 Japan

**Keywords:** Bidirectional shunt, Patent foraman ovale, Off-pump coronary artery bypass grafting, Hypoxemia, Postoperative management

## Abstract

**Background:**

Interatrial right-to-left shunt flow through a patent foramen ovale (PFO) can be caused by changes in heart position for anastomosis during off-pump coronary artery bypass (OPCAB). We herein present a case in which the direction of PFO shunt flow changed with heart position during OPCAB and the ventilation settings after sternal closure.

**Case presentation:**

A 66-year-old man with interstitial pneumonia underwent OPCAB. Preoperative transesophageal echocardiography revealed right-to-left shunt flow through a PFO induced by the Valsalva maneuver. During OPCAB, heart displacement resulted in right-to-left shunting and acute hypoxemia, which quickly improved with increase of inspired oxygen fraction. After chest closure, bidirectional shunt flow developed under increased airway pressure.

**Conclusions:**

Vigilant intraoperative monitoring with TEE and postoperative airway pressure management are important to address shunt flow and hypoxemia due to PFO.

**Supplementary Information:**

The online version contains supplementary material available at 10.1186/s40981-024-00748-7.

## Background

Patent foramen ovale (PFO) results from incomplete anatomical closure of the foramen ovale during infancy [1] and persists in 27.3% of adults [2]. It is well known that right-to-left shunt flow through a PFO can cause hypoxemia during off-pump coronary artery bypass (OPCAB) [3–6]. However, how interatrial pressure gradients change due to surgical manipulations and airway pressure, and how these changes affect the direction of the shunt flow has rarely been reported.

Herein, we report the case of a patient who developed a right-to-left shunt through a previously undiagnosed PFO and exacerbated hypoxemia due to heart displacement during OPCAB. Transesophageal echocardiography (TEE) using color flow Doppler revealed a marked right-to-left shunt flow through the PFO. The shunt flow disappeared when the heart returned to its original position after coronary artery anastomosis; however, after chest closure, increased airway pressure by adjusting the positive end-expiratory pressure (PEEP) level caused the shunt flow to become bidirectional, a novel finding we aim to highlight in this case report.

## Case presentation

A 66-year-old man with hypertension, diabetes mellitus, and interstitial pneumonia developed acute heart failure 9 months prior to presentation and was diagnosed with 3-vessel coronary artery disease. Subsequently, OPCAB was performed.

**Fig. 1:**
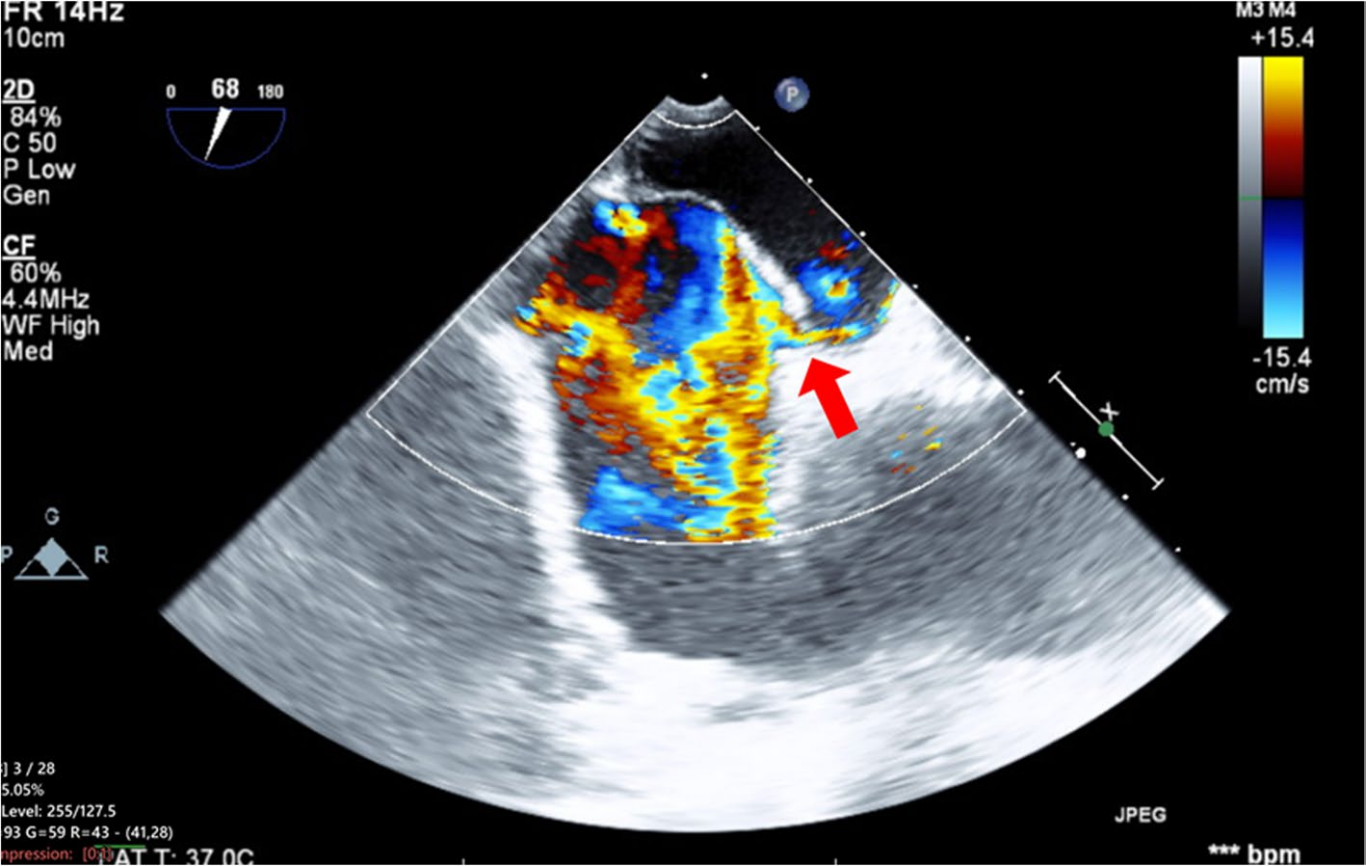
Midesophageal bicaval view during post-anesthesia induction Valsalva maneuver. Red arrow indicates right-to-left shunt flow through patent foramen ovale

General anesthesia was induced using intravenous midazolam, fentanyl, and rocuronium, and the patient was hemodynamically stable. In addition to standard monitoring, central venous pressure (CVP) was measured. To prevent acute exacerbation of interstitial lung disease, pressure-controlled ventilation was maintained with a low fraction of inspiratory oxygen (F_I_O_2_) at 35%, alongside low airway pressure set to an inspiratory pressure of 10 cmH_2_O and a PEEP of 5 cmH_2_O. Preoperative TEE showed a left ventricular ejection fraction of 56% with no regional wall motion abnormalities or significant valve dysfunction. Tricuspid regurgitation pressure gradient was 18.3 mmHg, which suggested pulmonary hypertension was not present. Midesophageal bicaval view revealed an atrial septal aneurysm, which suggested the coexistence of a PFO. However, as no shunt flow was observed probably due to the low CVP of 2 mmHg, we performed the Valsalva maneuver to detect the PFO. After releasing positive airway pressure, a right-to-left shunt was identified (Fig. 1, Video: Supplementary Material 1).

**Fig. 2:**
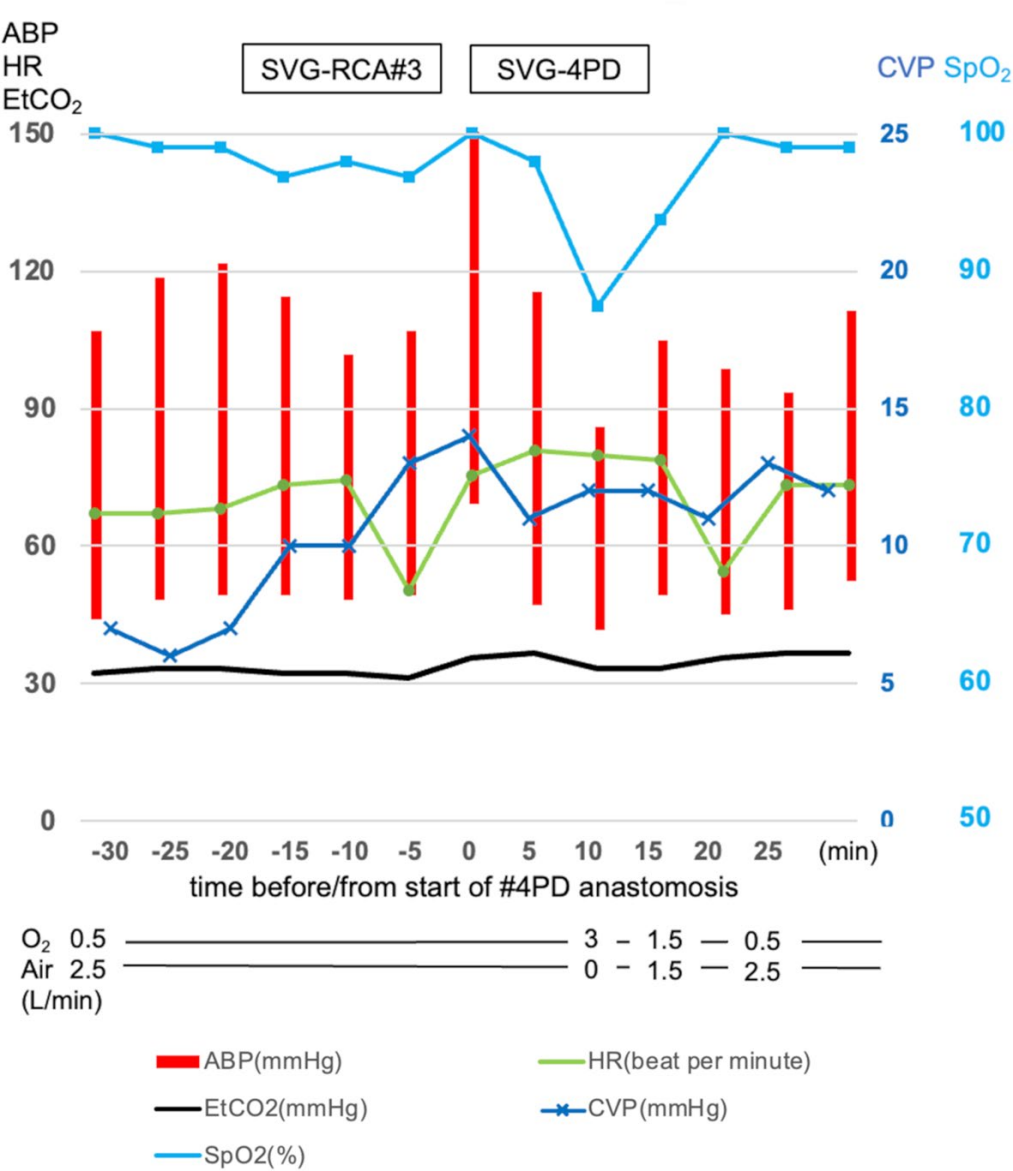
Hemodynamic trends during anastomosis of the saphenous vein graft to the posterior descending artery. Time 0 represents the initiation of the anastomosis. Note an abrupt decrease of peripheral oxygen saturation 10 min after starting anastomosis. Abbreviations: ABP, arterial blood pressure; CVP, central venous pressure; EtCO_2_, end-tidal carbon dioxide concentration; HR, heart rate; SpO_2_, peripheral capillary oxygen saturation; SVG-RCA#3, the saphenous vein graft to the right coronary artery #3; SVG-4PD, the saphenous vein graft to the posterior descending artery anastomosis

**Fig. 3:**
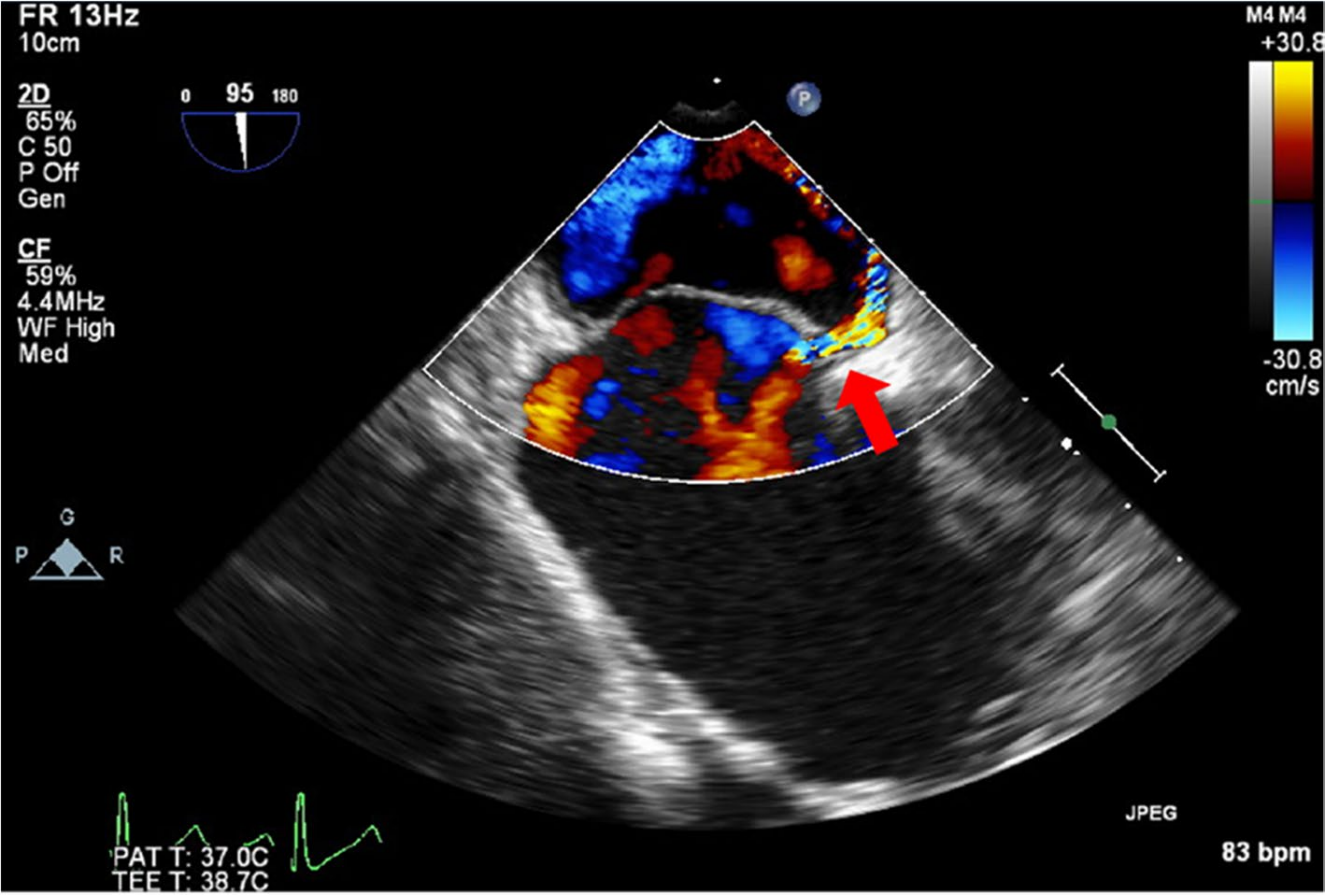
Midesophageal bicaval view during anastomosis of the saphenous vein graft to the posterior descending artery. Red arrow indicates right-to-left shunt flow through patent foramen ovale (increased from the preoperative flow presented in Fig. 1.)

After median sternotomy and pericardiectomy, in situ grafts of bilateral internal thoracic artery and a free saphenous vein graft (SVG) were harvested. After heparinization, the proximal end of the SVG was anastomosed firstly to the aortic wall by aortic side clamping. Subsequently, the left internal thoracic artery graft was anastomosed to the left anterior descending artery, and the right internal thoracic artery graft to the posterolateral artery. The heart was lifted in order to visualize the target vessels, which had minimum effects on hemodynamics. The distal anastomosis of the SVG to the right coronary artery was performed sequentially. The patient was placed in the Trendelenburg position. When the heart was elevated for the anastomosis of the SVG to the right coronary artery #3, CVP increased to 13 mmHg. The peripheral arterial oxygen saturation (SpO_2_) remained around 97% during the anastomosis. Next, for the distal anastomosis of the SVG to the posterior descending artery, the apex was displaced vertically, resulting in ventricular compression and deformation, which led to a sudden decrease in SpO_2_ from 98 to 88% (Fig. 2). No airway or ventilation problems were observed. Heart rate and blood pressure were maintained; CVP was still elevated at 14 mmHg (8 mmHg before heart displacement) and end-tidal carbon dioxide concentration (EtCO_2_) decreased slightly from 42 to 38 mmHg. TEE demonstrated an increased right-to-left shunt flow through the PFO compared to preoperative induction (Fig. 3, Video: Supplementary Material 2). Tricuspid regurgitation was absent on TEE. F_I_O_2_ increased to 100% and swiftly restored SpO_2_ to 98% within a minute, without altering the heart position. The surgeon completed the anastomosis; the heart position was returned to normal, and the shunt flow disappeared. After consulting with the surgeons, the decision was made not to close the PFO as shunt flow was not apparent in the normal heart position.

**Fig. 4:**
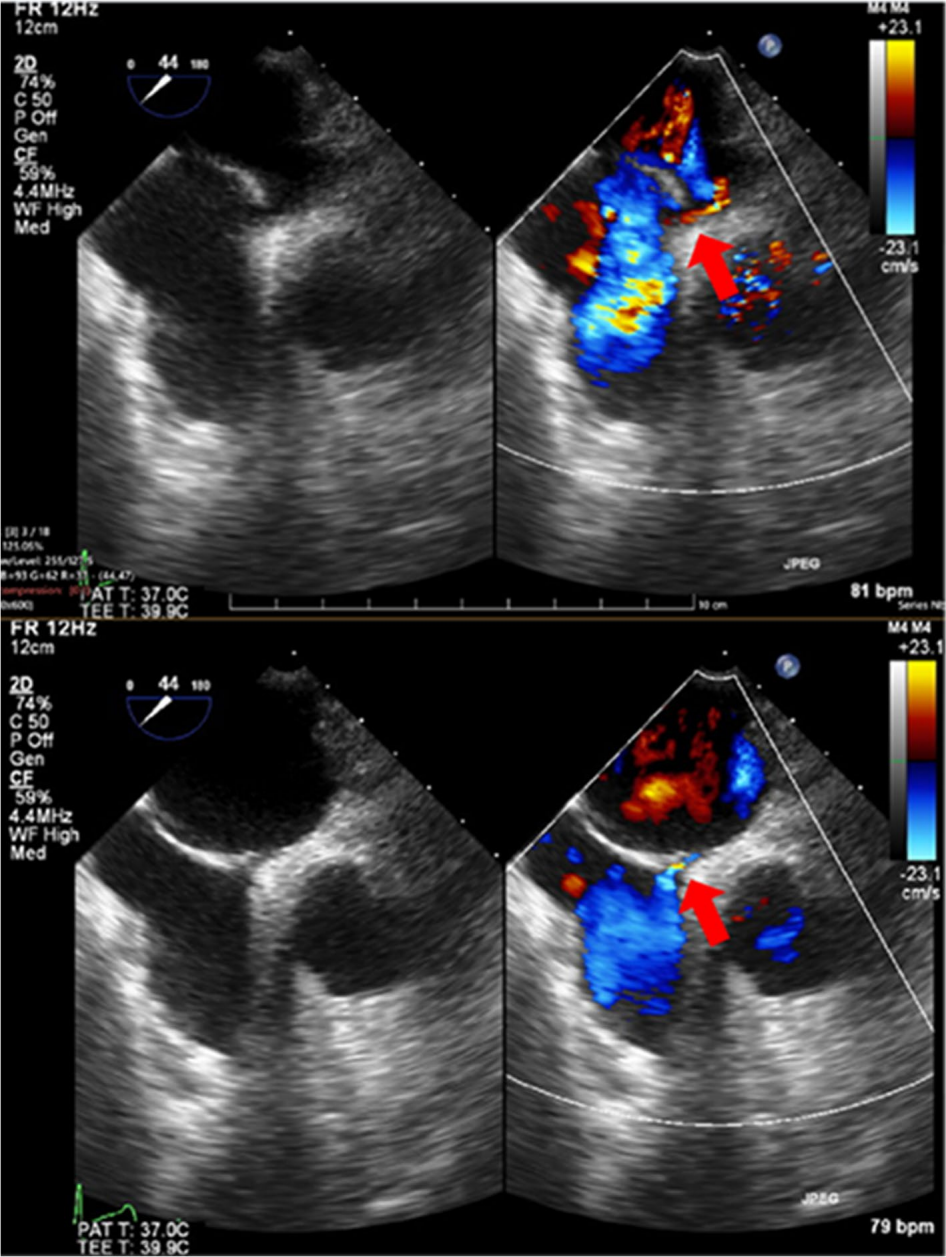
Midesophageal bicaval view post-sternum closure under high airway pressure. A. Red arrow indicates right-to-left shunt flow through patent foramen ovale during the expiratory phase of mechanical ventilation. B. Red arrow indicates left-to-right shunt flow through patent foramen ovale during the inspiratory phase of mechanical ventilation

Following sternal closure, ventilation settings were altered to increase airway pressure (inspiratory pressure, 15 cmH_2_O; PEEP, 10 cmH_2_O) on a trial basis for postoperative ventilation management, leading to a corresponding increase in CVP to 11 mmHg and the appearance of bidirectional shunt flow through the PFO (Fig. 4, Video: Supplementary Material 3). SpO_2 _was maintained at 95% during this period with an F_I_O_2_ of 0.48. Shunt flow through the PFO was absent at a lower airway pressure (inspiratory pressure, 10 cmH_2_O; PEEP, 5 cmH_2_O) and a CVP of 9 mmHg. In the intensive care unit, ventilation settings were adjusted to prevent high airway pressure, and the patient was extubated 8 h after surgery. The patient was discharged 12 days after the surgery without any complications.

## Discussion

The direction of shunt flow through the PFO is determined by the pressure gradient between the left and right atria. Shunt flow does not always occur through the PFO. As the left atrial pressure (LAP) is usually marginally exceeds right atrial pressure (RAP), most small PFOs are functionally closed and clinically insignificant. However, during the perioperative period, hemodynamic changes can considerably affect the delicate pressure balance [5, 6].

The Valsalva maneuver allows for detecting PFO with higher sensitivity [7]. This maneuver increases intrathoracic pressure, decreasing blood return from the systemic circulation to the right atrium. When pressure is released, venous return surges, elevating RAP above LAP, potentially causing a right-to-left shunt flow through the PFO [8, 9].

Surgical repositioning of the heart during OPCAB can also alter atrial pressure (Table 1). Anastomosis of the posterior descending artery is typically performed by lifting the apex of the heart using a heart positioner. This maneuver compresses both ventricles and disrupts diastolic filling, resulting in an increase in both RAP and LAP, with a more pronounced rise in RAP compared to LAP [10]. These changes can reverse the pressure gradient between RAP and LAP, leading to a right-to-left shunt through the PFO. Several case reports document the appearance of right-to-left shunt during anastomosis of the right coronary artery [3–5]. The extent of heart displacement varies with surgical factors (anastomotic location, graft length, surgeon preference, etc.), potentially influencing the appearance and direction of shunt flow [11]. In the present case, hypoxemia resulting from the right-to-left shunt was resolved with increasing F_I_O_2_, suggesting a relatively small shunt flow fraction. The degree of hypoxemia due to right-to-left shunt flow through the PFO depends on the volume of shunt blood flow. When shunt blood flow is significant, increasing F_I_O_2_is less effective in improving arterial oxygenation [12]. In such cases, it may be necessary to perform coronary artery bypass grafting under cardiopulmonary bypass or to close the PFO under cardiac arrest [4].

**Table 1:** Causes of right-to-left shunt via PFO and possible effects to atrial pressures

	RAP	LAP
Cardiac displacement during coronary anastomosis	↑↑ RV compression by heart stabilizer / positioner	↑ Impaired LV diastolic filling
Increased airway pressure	↑ Increased intrathoracic pressure	↓ Decreased venous return

*RAP* Right atrial pressure, *LAP* Left atrial pressure, *RV* Right ventricle, *LV* Left ventricle

Notably, in the present case, bidirectional flow was observed under high intrathoracic pressure (inspiratory pressure, 15 cmH2O; PEEP, 10 cmH_2_O), despite the absence of shunting at lower intrathoracic pressure (inspiratory pressure, 10 cmH_2_O; PEEP, 5 cmH_2_O). These changes in shunt blood flow have not been reported in other case reports. In a study of patients undergoing noncardiac surgery under general anesthesia, who were gradually increased to a PEEP of 19 cmH_2_O during mechanical ventilation, TEE examination revealed a right-to-left shunt through the PFO that appeared at a PEEP of 10 cmH_2_O or higher, but was not seen at 0 or 5 cmH_2_O [13]. This finding is consistent with the present case, indicating that high PEEP levels around 10 cmH_2_O can potentially reverse the pressure gradient between RAP and LAP. An increase in intrathoracic pressure would elevate RAP, while a decrease in venous return would reduce blood filling to the left ventricular system, thereby decreasing LAP (Table 1). Although LAP was not measured, we speculated that a right-to-left shunt appeared in this patient when the mean CVP (= RAP) during right coronary artery anastomosis was 14 mmHg, which substantially exceeded the LAP. In addition, when CVP is less than 9 mmHg during normal heart position, LAP is always greater than RAP, so no shunt occurred. When the airway pressure is increased and CVP elevated to 11 mmHg, RAP and LAP became almost equal, and a bidirectional shunt appeared because the pressure difference between RAP and LAP can easily reverse during the cardiac cycle. On TEE examination, it appears that a right-to-left shunt occurs during systole and a left-to-right shunt occurs during diastole, but it is unclear why the interatrial pressure gradient changed during the cardiac cycle in this way.

In conclusion, we encountered a patient with a right-to-left shunt through the PFO detected by TEE, which resulted in hypoxemia exacerbated by cardiac displacement during OPCAB, and shunt direction dynamically changed with high airway pressure after chest closure. It is important to recognize that in patients who have a PFO, surgical manipulation or positive pressure ventilation can lead to development of a right-to-left shunt and hypoxemia. Whenever an event such as cardiac displacement or increased airway pressure occurs, assessing the direction and volume of the shunt with TEE is essential. Furthermore, high airway pressure during mechanical ventilation should be avoided to prevent the development of a right-to-left shunt and hypoxemia.

## Supplementary Information

Supplementary Material 1.

Supplementary Material 2.

Supplementary Material 3.

## Data Availability

Not applicable.

## Abbreviations

CVP: Central venous pressure
EtCO_2_: End-tidal carbon dioxide concentration
F_I_O_2_: Inspired oxygen fraction
LAP: Left atrial pressure
OPCAB: Off-pump coronary artery bypass grafting
PEEP: Positive end-expiratory pressure
PFO: Patent Foramen Ovale
RAP: Right atrial pressure
SpO_2_: Peripheral capillary oxygen saturation
SVG: Saphenous vein graft;
TEE: Transesophageal echocardiography
